# Phase II study of Radium‐223 dichloride combined with hormonal therapy for hormone receptor‐positive, bone‐dominant metastatic breast cancer

**DOI:** 10.1002/cam4.2780

**Published:** 2019-12-18

**Authors:** Naoto T. Ueno, Rie K. Tahara, Takeo Fujii, James M. Reuben, Hui Gao, Babita Saigal, Anthony Lucci, Toshiaki Iwase, Nuhad K. Ibrahim, Senthil Damodaran, Yu Shen, Diane D. Liu, Gabriel N. Hortobagyi, Debu Tripathy, Bora Lim, Beth A. Chasen

**Affiliations:** ^1^ Department of Breast Medical Oncology The University of Texas MD Anderson Cancer Center Houston TX USA; ^2^ Department of Hematopathology The University of Texas MD Anderson Cancer Center Houston TX USA; ^3^ Department of Breast Surgical Oncology The University of Texas MD Anderson Cancer Center Houston TX USA; ^4^ Department of Biostatistics The University of Texas MD Anderson Cancer Center Houston TX USA; ^5^ Department of Nuclear Medicine The University of Texas MD Anderson Cancer Center Houston TX USA

**Keywords:** alphatherapy, bone metastasis, breast cancer, hormonal therapy, hormone receptor‐positive, Radium‐223 dichloride

## Abstract

**Background:**

Radium‐223 dichloride (Ra‐223) is a targeted alpha therapy that induces localized cytotoxicity in bone metastases. We evaluated the efficacy and safety of Ra‐223 plus hormonal therapy in hormone receptor‐positive (HR+), bone‐dominant metastatic breast cancer.

**Methods:**

In this single‐center phase II study, 36 patients received Ra‐223 (55 kBq/kg intravenously every 4 weeks) up to 6 cycles with endocrine therapy. The primary objective was to determine the clinical disease control rate at 9 months. Secondary objectives were to determine (a) tumor response rate at 6 months, (b) progression‐free survival (PFS) durations, and (c) safety.

**Results:**

The median number of prior systemic treatments for metastatic disease was 1 (range, 0‐4). The disease control rate at 9 months was 49%. The tumor response rate at 6 months was 54% (complete response, 21%; partial, 32%). The median PFS was 7.4 months (95% CI, 4.8‐not reached [NR]). The median bone‐PFS was 16 months (95% CI, 7.3‐NR). There were no grade 3/4 adverse events.

**Conclusions:**

Ra‐223 with hormonal therapy showed possible efficacy in HR+ bone‐dominant breast cancer metastasis, and adverse events were tolerable. We plan to further investigate the clinical application of Ra‐223 in these patients. (NCT02366130).

## Graphical abstract

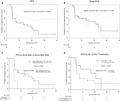

## 1 INTRODUCTION

Bone is the most common site of metastasis, especially in hormone‐receptor‐positive (HR+) breast cancers; bone metastasis occurs in 65%‐75% of patients with metastatic breast cancer.^1^, ^2^ Among patients with bone metastasis, approximately 20% of patients have bone‐only disease.^1^, ^2^, ^3^, ^4^ These metastases can cause skeletal‐related events, leading to reduced quality of life. For bone metastasis, bisphosphonates and denosumab have been proven to reduce the incidence of skeletal‐related events.^5^, ^6^, ^7^ Strontium‐89 and samarium‐153 has been used as radiotherapy for palliation of pain caused by bone metastasis^8^; however, there has been no approved antitumoral agent in breast cancer for treatment of bone metastasis.

Radium‐223 dichloride (Ra‐223) is a targeted alpha therapy that has been shown in preclinical studies to target bone metastases.^9^, ^10^ The alpha particles emitted from Ra‐223 lead to a localized cytotoxic effect to bone metastases.^9^, ^11^ Ra‐223 inhibits cancer growth by dual targeting of not only cancer cells but also the bone microenvironment.^12^ Ra‐223 is FDA approved for patients with castration‐resistant prostate cancer with bone metastasis, and in that patient population has shown significant improvement in overall survival (OS) compared with placebo (median OS, 14.9 months vs 11.3 months; hazard ratio, 0.70; *P* < .001).^13^ A phase II study of Ra‐223 in patients with bone‐dominant metastatic breast cancer who were no longer benefiting from hormonal therapy showed metabolic response in bone metastases measured by ^18^F‐fluorodeoxyglucose positron emission tomography/computed tomography (FDG‐PET/CT).^14^

Here, we present the result of a single‐center phase II study evaluating the efficacy and safety of combined Ra‐223 and hormonal therapy in patients with HR+, bone‐dominant metastatic breast cancer.

## 2 PATIENTS AND METHODS

### 2.1 Study design

This single‐arm, open‐label phase II trial (ClinicalTrials.gov identifier: NCT02366130) was approved by the Institutional Review Board at The University of Texas MD Anderson Cancer Center (protocol number: 2014‐0508). The study was conducted in accordance with the principles of the Declaration of Helsinki and Good Clinical Practice. All patients signed informed consent forms upon enrollment in the study. Patients received Ra‐223 (55 kBq/kg body weight, the standardized dose established by the US National Institute of Standards and Technology in 2015) as a bolus intravenous (IV) injection (up to 1 minute) every 4 weeks up to 6 cycles. Patients also received a hormonal agent—either tamoxifen, an aromatase inhibitor, or fulvestrant—at standard dosage and a subcutaneous injection of denosumab (120 mg every 4 weeks), which together were considered standard of care during the study period.

### 2.2 Eligibility

Eligibility criteria included age of at least 18 years and pathologically confirmed HR+ invasive breast cancer with metastases to 2 or more bones and/or the bone marrow. A concurrent single visceral metastasis smaller than 2 cm was allowed. There was no limit in the number of prior hormonal agents received, and one prior chemotherapy regimen in the metastatic setting was allowed. Patients with a single bone lesion, two or more visceral metastases, brain metastases, imminent spinal cord compression, and impending fracture were not eligible.

### 2.3 End points

The primary objective was to determine the disease control rate at 9 months, defined as the rate of the patients at that time with clinically complete or partial response or stable disease. We had three secondary objectives: (a) We determined the tumor response rate at 6 months, defined as the rate of the patients at that time with complete or partial response using the Positron Emission Tomography Response Criteria in Solid Tumors (PERCIST).^15^ PERCIST is an adaptation of the most widely used criteria for evaluation of cancer response, Response Evaluation Criteria in Solid Tumors (RECIST). RECIST is based on the anatomical measurement of solid tumors using conventional imaging or calipers; however, it is often difficult to measure the size of tumors within bone. In contrast, PERCIST is based on measurement of tumors using FDG‐PET/CT, allowing anatomical and metabolic assessment in tandem.^15^, ^16^, ^17^, ^18^ FDG‐PET/CT provides a measure of standardized uptake value (SUV), which reflects tumor glucose metabolism and tumor aggressiveness. We compared SUVs for the hottest single lesion at each time point. (b) We determined progression‐free survival (PFS) and bone‐PFS durations. PFS was defined as the duration from the first day of Ra‐223 injection to either the detected date of any disease progression or the date of the last scan without progression. Bone‐PFS was defined as the duration from the first day of Ra‐223 injection to either the detected date of bone disease progression or the date of the last scan without bone progression. (c) We determined the safety of the treatment combination by assessing AEs based on NCI‐CTCAE v4.03.

Exploratory objectives were to determine the association between survival outcomes and the proportions of epithelial circulating tumor cells (CTCs) and CTCs undergoing epithelial‐mesenchymal transition (EMT‐CTCs). CTCs were enumerated using the FDA‐approved CellSearch System (Menarini Silicon Biosystems, Inc). EMT‐CTCs were detected using the AdnaTest EMT‐2 kit (QIAGEN).

### 2.4 Assessments

All patients who received at least one dose of Ra‐223 were included in the efficacy analysis. Efficacy was clinically evaluated with bone scan and FDG‐PET/CT at 6 and 9 months compared with baseline. Efficacy outcome according to PERCIST was evaluated at 6 and 9 months. We used RECIST 1.1. to assess response of measurable visceral lesions. Safety assessment was performed before each cycle of Ra‐223. CTC counts and presence of EMT‐CTCs were measured using peripheral blood samples (7.5 mL for CTCs and 5.0 mL for EMT‐CTCs) collected at baseline, 6 and 9 months.

### 2.5 Statistical analysis

The 9‐month timeframe was chosen because our group reported that the median PFS duration for patients with HR+ bone‐only metastatic breast cancer is 12 to 18 months. Those data suggest that there will be no progression of disease in about 70% of patients at 9 months.^1^ We estimated that with 36 patients, we would have 85% power to detect a disease control rate of 90% against 70% based on our previous report with a two‐sided exact binomial test at a significance level of 5%.^1^ Time‐to‐event outcomes were estimated using the Kaplan‐Meier method, and the log‐rank test was used to compare two groups of interest. Patient characteristics and toxicity data were summarized using standard descriptive analysis.

## 3 RESULTS

### 3.1 Patient characteristics

**Figure 1:**
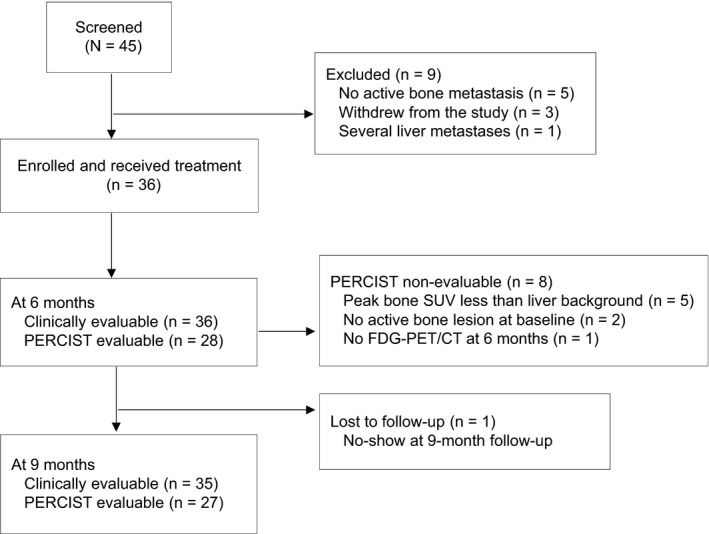
CONSORT flow diagram

From March 2015 through December 2017, 45 patients were accrued. Nine patients were determined to be ineligible; five patients were found to not have active bone metastasis, three patients withdrew from the study before the start of cycle 1, and one patient was found to have several liver metastases (Figure 1). Baseline characteristics for the remaining 36 patients are shown in Table 1. The median age of the patients was 58 years (range 31‐79). Thirty‐two patients (89%) had only bone metastasis, and four patients (11%) had both bone and another site of metastasis (3 patients: lymph node; 1 patient: liver). The median number of prior systemic therapy regimens received in the metastatic setting was 1 (range, 0‐4); the types of systemic therapy were hormonal therapy in 23 patients (64%), chemotherapy in six patients (17%), and cyclin‐dependent kinase (CDK) 4/6 inhibitors in 10 (28%) patients. The hormonal agents that were given with Ra‐223 were tamoxifen (8 patients), an aromatase inhibitor (21 patients), and fulvestrant (7 patients). There were 18 patients (50%) who received prior radiation therapy to bone metastases, but no prior radiated lesions were included within the Ra‐223 targeted bone metastases.

**Table 1:** Baseline characteristics of the 36 patients who received treatment

Characteristic	Patients (N = 36)
Age, y	
Median	58
Range	31‐79
Menopausal status, no. (%)	
Premenopausal	11 (31)
Postmenopausal	25 (69)
ECOG performance status, no. (%)	
0	28 (78)
1	8 (22)
HER2 status, no. (%)	
Negative	35 (97)
Positive	1 (3)
De novo metastasis, no. (%)	
Yes	8 (22)
No	28 (78)
Metastatic sites, no. (%)	
Bone only	32 (89)
Bone with another site	4 (11)
Lymph node	3 (8)
Liver	1 (3)
Disease‐free interval from diagnosis to first metastasis, years	
Median	3
Range	0‐21
Time from first metastasis to study entry, months	
Median	7
Range	0‐121
Prior systemic therapy for metastatic disease, no. (%)	
No prior systemic therapy	12 (33)
Hormonal therapy	23 (64)
1 line	15 (42)
2 lines	5 (14)
3 lines	2 (6)
4 lines	1 (3)
Chemotherapy	6 (17)
CDK4/6 inhibitor	10 (28)
Radiation to bone metastasis	18 (50)

### 3.2 Efficacy

**Figure 2:**
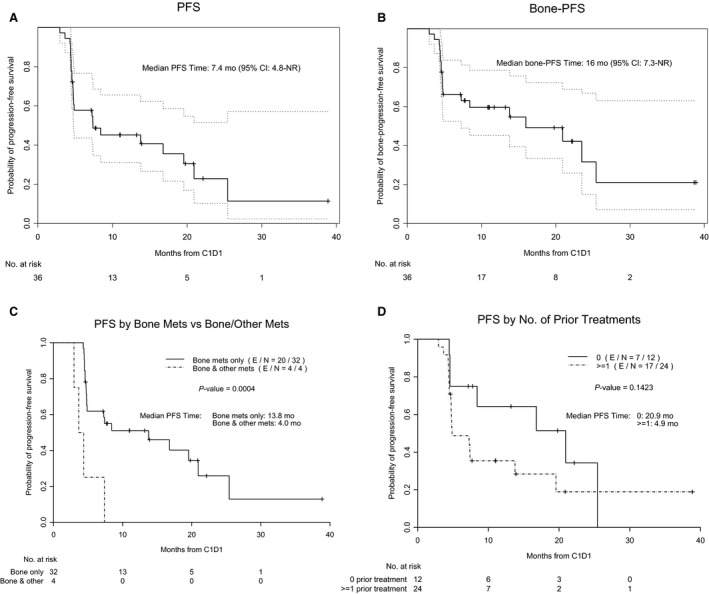
Progression‐free survival (PFS) for the 36 patients who received treatment. Median follow‐up time for progression was 19.8 months (95% confidence interval [CI]: 11—not reached [NR]; range: 4.6‐38.9). A, PFS for all patients (N = 36) from the first day of Ra‐223 injection (cycle 1, day 1; C1D1). B, Bone‐PFS. C, PFS by site of metastasis (bone only vs bone plus other metastasis). D, PFS by number of prior treatment regimens (no prior treatment vs at least one prior treatment regimen). Dotted lines represent 95% CI. E/N, Number of patients with disease progression/ Total number of evaluable patients

The median follow‐up time was 19.8 months. One patient was lost to follow‐up between 6 and 9 months. The median number of Ra‐223 injections was 5.5 (range, 3‐6). The disease control rate, defined as the percentage of patients who had a complete, partial, or stable clinical response, was 49% at 9 months (Table 2). FDG‐PET/CT data enabling PERCIST evaluation were available for 28 patients at 6 months. Response was not evaluable by PERCIST in eight patients: peak SUV of the most active bone lesion was less than the minimum comparison SUV of the liver background at baseline in five patients; there was no active bone lesion by FDG‐PET/CT at baseline in two patients; and FDG‐PET/CT was not performed at 6 months in one patient. The tumor response rate, defined as the percentage of patients who had a complete or partial response using the PERCIST criteria, was 54% at 6 months (6 patients with complete response). The peak SUV of the hottest single lesion on FDG‐PET/CT decreased significantly from the baseline value to those at 6 and 9 months; the median decreases were 2.0 (*P* = .0032) at 6 months and 2.5 (*P* = .0002) at 9 months (supplementary Table). The median PFS time was 7.4 months (95% confidence interval [CI], 4.8‐NR) (Figure 2A). The median bone‐PFS was 16 months (95% CI, 7.3‐NR) (Figure 2B). The median PFS was significantly longer in patients with bone metastasis only than in those with bone and another site of metastasis at baseline (13.8 vs 4.0 months; *P* = .0004) (Figure 2C). Patients who had not had prior systemic treatment (N = 12, 33%) tended to have longer median PFS durations than did those who had had at least one prior systemic treatment (N = 24, 67%), but the difference was not statistically significant (20.9 vs 4.9 months; *P* = .1423) (Figure 2D). Among the 18 patients with progressive disease at 9 months, disease progression was seen in the following metastatic sites: bone only (N = 7, 39%), bone and liver (N = 5, 28%), liver only (N = 4, 22%), bone and chest (N = 1, 6%), and liver and lung (N = 1, 6%). There was also an indication that a higher suppression in SUV at 6 months and 9 months may be associated with a longer time to progression (supplementary Figure S1).

**Table 2:** Disease control and tumor response rates for the patients who received treatment and were evaluable at the indicated time points

Response	N (%)	
Disease control (clinical)	6 mo (N = 36)	9 mo (N = 35)
Complete, partial, or stable response	21 (58)	17 (49)
Progressive disease	15 (42)	18 (51)
Tumor response (PERCIST)	6 mo (N = 28)	9 mo (N = 27)
Complete response	6 (21)	9 (33)
Partial response	9 (32)	3 (11)
Stable disease	1 (4)	1 (4)
Progressive disease	12 (43)	14 (52)

### 3.3 Safety

There were no grade 3 or 4 AEs. AEs that occurred in at least 4 of the 36 patients are summarized in Table 3. The major nonhematological AEs were bone pain (78% of patients), fatigue (42%), nausea (36%), aspartate aminotransferase (AST)/alanine aminotransferase (ALT) elevation (31%), and diarrhea (31%). The hematologic AEs were lymphocytopenia (28%), neutropenia (25%), anemia (17%), and thrombocytopenia (11%). There was no treatment delay or discontinuation due to AEs.

**Table 3:** Adverse events occurring in at least 4 of the 36 patients who received treatment

Adverse event	All Grades N (%)	Grade 1 N	Grade 2 N
Nonhematologic			
Bone pain	28 (78)	20	8
Fatigue	15 (42)	5	10
Nausea	13 (36)	8	5
AST/ALT elevation	11 (31)	9	2
Diarrhea	11 (31)	8	3
Headache	7 (19)	4	3
Hyperglycemia	7 (19)	6	1
Hypomagnesemia	6 (17)	5	1
Flu‐like symptoms	6 (17)	5	1
Hot flashes	5 (14)	4	1
Arthralgia	4 (11)	3	1
Insomnia	4 (11)	4	0
Hematologic			
Lymphocytopenia	10 (28)	8	2
Neutropenia	9 (25)	2	7
Anemia	6 (17)	5	1
Thrombocytopenia	4 (11)	4	0

Abbreviations: ALT, alanine aminotransferase; AST, aspartate aminotransferase.

### 3.4 CTCs and EMT‐CTCs

At least 1 CTC was found in 27 patients (75%) at baseline. The median CTC count at baseline was 4 (range, 0‐306) per 7.5 mL of peripheral blood. Patients with <5 CTCs at baseline (N = 20, 56%) tended to have longer median PFS durations than did those with ≥5 CTCs (N = 16, 44%) (16.8 vs 4.8 months) (*P* = .1486) (Figure S2A). In patients with bone metastasis only, those with lower CTC count at baseline (<5 CTCs) tended to have longer time to progression (16.8 vs 4.9 months) (*P* = .18) (Figure S2D). We also compared PFS in patient groups divided using cutoffs of 1 CTC and 2 CTCs at baseline; lower CTC counts at baseline were significantly associated with better outcome (Figures S2B,C,E,F).

Only four patients (11%) were positive for EMT‐CTCs at baseline. Baseline EMT‐CTC status was not associated with response or PFS.

## 4 DISCUSSION

In patients with HR+, bone‐dominant metastatic breast cancer, Ra‐223 showed a high disease control rate at 9 months (49%) and tumor response rate at 6 months (54%) with tolerable AEs. A median PFS of 7.4 months was observed despite the fact that more than half of the patients had had prior hormonal treatment, almost 20% of patients had had prior chemotherapy, and 30% of patients had previously received a CDK4/6 inhibitor for metastatic disease. We found that patients with bone‐only metastasis at baseline showed significantly longer PFS than those with bone metastasis and a single lesion smaller than 2 cm in another metastatic site at baseline (median 13.8 months vs 4.0 months; *P* = .0004). The efficacy of Ra‐223 combination therapy was thus more prominent against bone lesions compared with metastases outside the bone.

The overall PFS of 7.4 months compares favorably with those of two key clinical trials that combined hormonal agents with CDK4/6 inhibitors; the latter are key agents in HR+ breast cancer as both first‐line systemic treatment and second‐line or subsequent treatment for hormonal‐therapy‐refractory disease. PALOMA‐3, a randomized phase III clinical trial testing CDK4/6 inhibitor palbociclib combined with hormonal agent fulvestrant in premenopausal women with HR+ advanced breast cancer that progressed during previous hormonal therapy, showed a similar median PFS, 9.5 months.^19^ MONALEESA‐2, a randomized phase III clinical trial testing the efficacy of CDK4/6 inhibitor ribociclib with hormonal agent letrozole in patients with HR+/HER2‐ advanced breast cancer, demonstrated a PFS of 25.3 months^20^; however, that trial was conducted as first‐line treatment, whereas in our study more than half of the patients had received previous hormonal therapy in the metastatic setting.

The safety profile we observed was similar to the result of a phase III trial of Ra‐223 in castration‐resistant prostate cancer with bone metastases.^13^ Our results support the safety and tolerability of Ra‐223 in combination with hormonal therapy. There were no CTCAE grade 3 or 4 AEs, and there was no delay or discontinuation of Ra‐223 due to AEs. Ra‐223 did not adversely affect bone marrow function. Not having grade 3 and 4 AEs is extremely important, especially in patients with bone‐only metastasis because these patients tend to have long survival and could be on systemic treatment for many years.^4^

We also evaluated whether baseline counts of CTCs and EMT‐CTCs were associated with survival outcome. CTCs, cancer cells circulating in the peripheral blood, have been shown to be detectable in 50% to 70% of patients with metastatic breast cancer.^21^, ^22^ CTCs are an independent prognostic factor in breast cancer metastasis, and PFS and OS have been shown to be superior among patients with fewer than 5 CTCs per 7.5 mL of peripheral blood.^21^, ^22^ In our study, patients with lower CTC count at baseline (<5 CTCs) tended to have a longer time to progression than did patients with ≥5 CTCs, although the difference was not statistically significant possibly due to small sample sizes. Cut‐offs of 1 CTC and 2 CTCs at baseline, which are usually used for evaluating nonmetastatic breast cancer,^23^, ^24^ were significantly associated with better outcome. EMT is one of the key mechanisms by which tumors acquire the traits needed to execute the multiple steps of metastasis.^25^ EMT‐CTC status at baseline was not associated with outcome. Patients with a low CTC count at baseline might have originally had good prognosis regardless of treatment, and it is difficult to assess how the addition of Ra‐223 contributed to this outcome.

To further investigate the clinical potential of Ra‐223 in breast cancer patients with bone metastasis, two randomized placebo‐controlled study with Ra‐223 vs placebo and hormonal therapy are ongoing. If such a study confirms that addition of Ra‐223 to hormonal therapy results in a prognosis superior to that of hormonal therapy alone, Ra‐223 will be an important drug for patients with bone‐dominant metastasis. There also may be potential exploratory avenues in combining Ra‐223 with chemotherapy and CDK4/6 inhibitors.

## 5 CONCLUSIONS

In summary, our study suggests that addition of Ra‐223 to a hormonal agent may provide a high disease control rate and be especially effective in controlling bone metastasis in patients with HR + bone‐dominant metastatic breast cancer, with minimal AEs. Additional studies are being planned to further investigate the clinical benefit of Ra‐223 in patients with bone‐dominant metastatic breast cancer.

## CONFLICT OF INTEREST

NT Ueno reports receiving clinical trial funding from Bayer AG and Amgen. All remaining authors have declared no conflicts of interest.

## AUTHOR CONTRIBUTIONS

Naoto. T. Ueno^:^ Conceptualization, data curation, formal analysis, funding acquisition, investigation, methodology, project administration, resources, software, supervision, validation, visualization, and writing—review and editing. Rie. K. Tahara: Data curation, formal analysis, investigation, resources, software, validation, visualization, writing—original draft, and writing—review and editing. Takeo. Fujii: Conceptualization, formal analysis, methodology, resources, software, supervision, validation, and writing—review and editing. James. M. Reuben, Anthony. Lucci: Conceptualization, data curation, formal analysis, investigation, methodology, project administration, supervision, validation, and writing—review and editing. Hui. Gao, Babita. Saigal, Toshiaki. Iwase: Data curation, investigation, and writing—review and editing. Nuhad. K. Ibrahim, Senthil. Damodaran: Investigation and writing—review and editing. Yu. Shen, Diane. D. Liu: Formal analysis, methodology, supervision, validation, visualization, and writing—review and editing. Gabriel. N. Hortobagyi, Debu. Tripathy, Bora. Lim: Investigation, supervision and writing—review and editing. Beth. A. Chasen: Conceptualization, data curation, formal analysis, investigation, methodology, supervision, validation, and writing—review and editing.

## PRECIS FOR USE IN THE TABLE OF CONTENTS

Radium‐223 dichloride is a targeted alpha therapy with localized cytotoxicity for bone metastasis. Our phase II trial which tested radium‐223 dichloride combined with hormonal therapy provided a high disease control with minimum toxicity in patients with hormone receptor‐positive, bone‐dominant metastatic breast cancer.

## ETHICS APPROVAL AND CONSENT TO PARTICIPATE

Investigator abide by Good Clinical Practice guidelines. Informed consent was obtained from all patients for this study. MD Anderson IRB approved this study. This study is under the guiding principles detailed in the Declaration of Helsinki.

## CONSENT FOR PUBLICATION

Consent for publication was obtained from all patients for this study.

## Data Availability

The data that support the findings of this study are available from the corresponding author upon reasonable request.
