# Supplementary material for: Phase II study of Radium‐223 dichloride combined with hormonal therapy for hormone receptor‐positive, bone‐dominant metastatic breast cancer
Source: Cancer Med. 2019 Dec 18;9(3):1025–32. doi: 10.1002/cam4.2780 (PMC6997080; doi:10.1002/cam4.2780)
Supplement: Supplementary file 1 [file CAM4-9-1025-s001.docx]

**Supplementary Table. Standardized uptake value (SUV) changes for the patients evaluable by PERCIST.**

**Figure S1. PFS by change in SUV at (A) 6 months and (B) 9 months.**

**Figure S2.** **PFS by baseline circulating tumor cell (CTC) count.**

**Supplementary Table. Standardized uptake value (SUV) changes for the patients evaluable by PERCIST.**

| **Variable** | **N** | **SUV** | | | | | **p-value*** |
| --- | --- | --- | --- | --- | --- | --- | --- |
|  |  | **Mean** | **SD** | **Median** | **Minimum** | **Maximum** |  |
| SUV at baseline | 28 | 4.8 | 1.8 | 4.3 | 2.7 | 9.2 |  |
| SUV at 6 months | 28 | 3.3 | 2.1 | 2.7 | 0.8 | 7.3 | 0.0032 |
| SUV at 9 months | 16 | 2.1 | 1.6 | 1.4 | 0.9 | 7.4 | 0.0002 |

SD, standard deviation.
*Signed-rank test comparing the SUV change to 0.

**Figure S1. PFS by change in SUV at (A) 6 months and (B) 9 months. The cutoff values were the median change at each time point.**

A

B

**Figure S2. PFS by baseline circulating tumor cell (CTC) count.** **A-C) PFS for all patients (N=36) compared based on a threshold of** **(A) 5 CTC, (B) 2 CTC, or (C) 1 CTC. D-F) PFS for patients with bone-only metastasis at baseline (N=32) compared based on a threshold of (D) 5 CTC, (E) 2 CTC, or (F) 1 CTC.**

A

B

C

D

E

F
